# Frequent Loss Expression of *Dab2* and Promotor Hypermethylation in Human Cancers: A Meta-Analysis and Systematic Review

**DOI:** 10.12669/pjms.302.4486

**Published:** 2014

**Authors:** Ziyin Zhang, Yihua Chen, JianJian Tang, Xuemei Xie

**Affiliations:** 1 Ziyin Zhang, Department of Neurosurgery, Meishan City People's Hospital, Meishan, Sichuan Province, 620010, China.; 2 Yihua Chen, Department of Pathology, People's Liberation Army General Hospital of Chengdu Military Region, Chengdu, Sichuan Province, 610083, China.; 3 JianJian Tang, Department of Neurosurgery, Meishan City People's Hospital, Meishan, Sichuan Province, 620010, China.; 4 Xuemei Xie, Department of Pathology, People's Liberation Army General Hospital of Chengdu Military Region, Chengdu, Sichuan Province, 610083, China.

**Keywords:** Dab2, Expression, Human, Cancers, Meta-Analysis

## Abstract

***Objective*** ***:*** Disabled-2 (Dab2) is an important endocytic adaptor which plays an inhibition role in cancer cell growth. The objective of this study was to systematically review expressions of Dab2 in human cancers.

***Methods*** ***:*** Eligible studies about Dab2 in human cancers were retrieved from databases of PubMed, Embase, Web of Science. Odds Ratios (ORs) with 95% confidence intervals (CIs) were calculated using Review Manager 5.0 software and statistical analyses were performed by the SPSS 13.0 software.

***Results*** ***:*** Fourteen case-control studies with a total of 689 human tumor tissues, 332 control tissues and 32 cancer cell lines were included in the meta-analysis study. The results indicated loss expressions of Dab2 were observed in 74.9% and 46.9% in human malignant cancer tissues and cancer cell lines, respectively. The ratio of *Dab2* promotor hypermethylation is 34.54% in cancer tissues which Dab2 expression are lost, but none in the control tissues or cells by Methylation-specific PCR (MSP).

***Conclusions*** ***:*** The expressions of Dab2 are frequently lost in human malignant cancer tissues, and promotor hypermethylation of Dab2 are common in human malignant cancer tissues, which is an important factor for the loss expression of Dab2 in human cancers tissues.

## INTRODUCTION

The gene *Disabled-2 (Dab2)* which also names DOC-2 (Differentially expressed in the Ovarian Carcinoma 2, DOC-2) owns two different splicing formats, and encodes two isoforms (p96-Dab2 and p67-Dab2).^1^^,^^2^ The main functional domain is the phosphotyrosine binding domain (PTB) of the N-terminal, which is a highly conserved sequence and plays a variety of functional roles in endocytosis, cell mitosis, and growth factor signaling.^3^^,^^4^ Especially, the p96-Dab2 is essential for the development of visceral endoderm during mouse embryogenesis and homologous with 93% full-length of mouse *Dab2*.^5^^-^^7^ Mechanistically, Dab2 is shown to bind with the growth factor receptor binding protein 2 (Grb2), consequently uncouple the activation of *c-Fos* expression and Ras/mitogen activated protein kinase (MAPK).^8^^-^^11^

Accumulated studies have shown that Dab2 is reduced or lost in human cancers, containing lung cancers, nasopharyngeal carcinomas, the breast cancers, and colorectal cancers, et al.^12^^-^^19^ Thus, it is gradually considered as a tumor suppressor gene. However, there are no complete credible studies to explain the concrete mechanisms, except a few of epigenetic studies about promoters or exons of *Dab2*.^16^^,^^17^^,^^19^

In the current study, we firstly conducted a meta-analysis of cohort studies to evaluate Dab2 expression level and its epigenetic variations in various human malignant cancers or cell lines. Furthermore, systematically investigated the concrete reasons for Dab2 expression loss, and its correlation with human oncogenesis.

## METHODS

***Literature*** ***search strategy: ***We adapted the Cochrane Central Register of Controlled Trials, and searched relevant papers published before September 31^st^ 2013 in Medline, Embase, Web of Science, and Scopus with relevant text words and medical subject headings that included all spellings of “*Disabled-2*’’ or “*DOC-2*’’ and ‘‘cancer’’ and ‘‘human’’. In addition, we firstly performed an initial scanning of the titles or abstracts, reference lists of retrieved papers and reviews to identify other potential relevant studies. Disputes were resolved through discussions among three authors.

***Criterions of Inclusion and Exclusion: ***We selected the eligible studies in the present meta-analysis using the following criterions: (1) use of an cohort study or case-control study design, and focusing on the correlations between Dab2 or DOC-2 and human cancers; (2) basic researches with big size of tissue samples. Studies were excluded using the following criterions: (1) not a case-control study or cohort study; (2) not a primary document, such as a letter, meta-analysis, review, duplicate or editorial article; (3) literatures with insufficient samples or incomplete data, and the conclusions are out of date.

***Data extraction*** ***: ***Three authors recorded the following details of each included research cooperatively, containing: authors, year of publication, the country of study, journal, materials and methods, study design, age of study population, pathological type of tumors, detecting sample size, source of participants, confounders adjusted for, effect sizes and 95% Confidence Index (CI) or standard errors of effect sizes. Differences were resolved by discussion among three authors in cases of conflicting evaluations.

***Statistical Analysis: ***The present case-control study was performed by Review Manager 5.0 software, the direct count method was used to estimate the expression level of Dab2 in human cancers. The odds ratios (OR) and 95% confidence intervals (CI) were computed by means of the Fisher’s exact probability test (two-tailed p-values). The data was analyzed by means of SPSS version 13.0 (SPSS Inc; Chicago, USA). This merged ORs and the 95% CI were obtained by means of the fixed or random effects model for each kind of human cancers. The heterogeneity was computed by Cochrans Q test, which P-value > 0.05 or *I*^2 ^> 50% indicated the existence of heterogeneity among studies. The subgroup analysis was used to explore sources of heterogeneity.

## RESULTS

**Fig.1:**
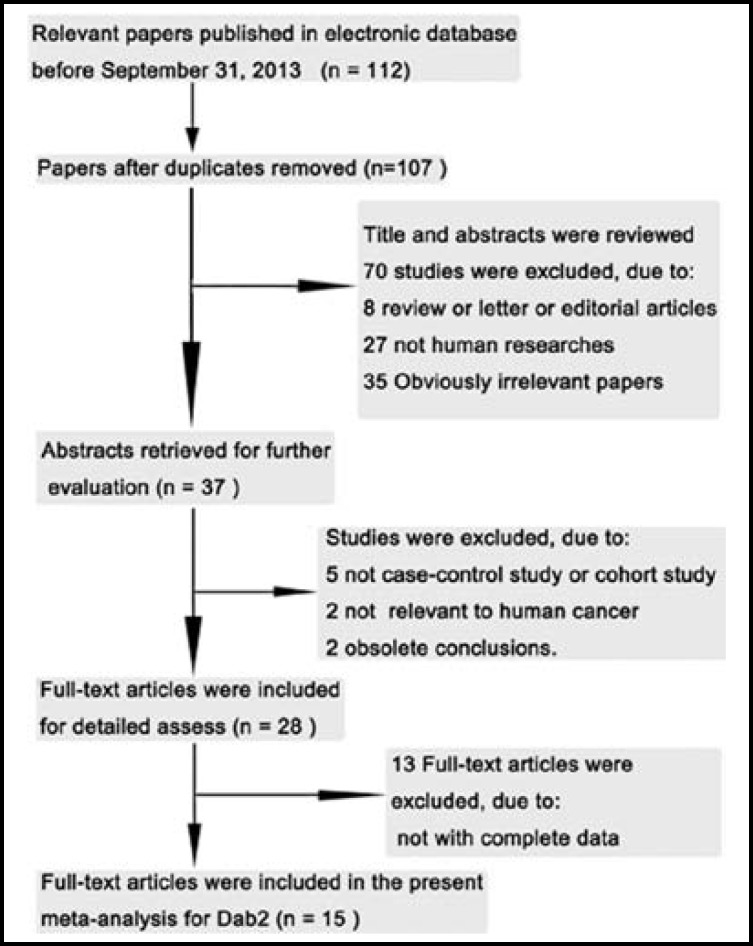
Flow Chart of Study Selection this meta-analysis

***Search of Eligible Studies:*** We initially retrieved 112 relevant papers in September, 2013. Finally, 15 studies were included in the present meta-analysis.Fig.1.

***Characteristics of Included Studies: ***The main characteristics of the 15 studies are presented in Table-I in which publication year ranges from 1998 to 2013^12^^-^^27^, and the quality scores vary from 5.5 to 7.5 points. A total of 395 different human cancer tissues and 32 cancer cell lines were utilized to analyze the expression loss of Dab2 by immunohistochemistry or western blot analysis. Furthermore, the analysis of aberrant hypermethylation of Dab2 promotor were performed in Nasopharyngeal carcinomas, Esophageal Squamous Cell Cancers, breast cancers and lung cancers, respectively.

**Fig.2:**
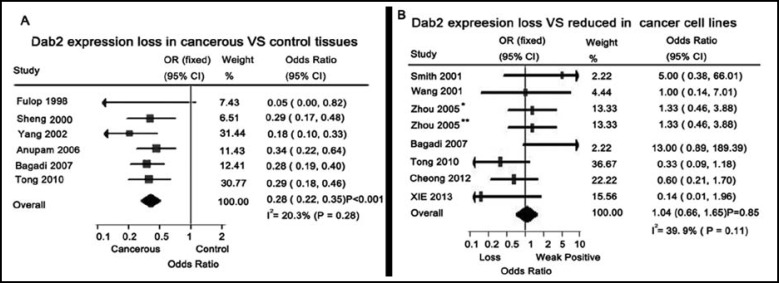
Forest plots of the meta-analysis of expressions of Dab2 in human cancers tissues and cell lines in current studies

***Frequent loss expression of Dab2 was observed in various human cancers tissues: ***The immunostained percentage of Dab2 expression were detected in five different kinds of human cancer tissues. Mok SC et al found that Dab2 expression were significantly down-regulated in ovarian cancers than normal ovarian tissues (the strong positive rate was 4.5% Versus 84.1%, and weak positive rate was 92.3% versus 0.0%).^12^ Analogously, Xu et al reported that Dab2 expression was significantly reduced in lung cancers than the non-cancerous tissues (the strong, moderate, and weak positive rate were 56.2% Versus 24.76%, 37.1% Versus 47.62% and 6.7% Versus 27.62%, respectively)^15^ Table-II. Furthermore, Tong et al found that Dab2 was un-detectable in 72% nasopharyngeal carcinomas of Chinese people.^16^ After pooling eligible data, absent expression of Dab2 were detected in 221 of 295 (74.9%) human malignant cancer tissues (OR = 0.28, 95% CI: 0.22 - 0.35, *P* < 0.001; I^2 ^= 20.3%, *P *_heterogeneity_ = 0.28) Fig.2A. Especially, XIE et al reported that the p96-Dab2 was expressed only in the nuclei of 31 cases (31/50, 62.0%) of normal lung tissues, and was lost in all the lung cancer tissues; which suggested that the two isoforms of Dab2 were differentially expressed in a tissue-specific manner.^14^

***The ectopic expressions of Dab2 were observed in several human malignant cancer*** ***cell*** ***lines: ***Data pooled from eight studies in this meta-analysis showed that the ectopic expression of Dab2 were observed in 17 human malignant cancer cell lines, including: A549, LTE, H1299, SH-SY5Y, HT1080, et al. Dab2 was un-detectable in MCF7, T47D, ZR-75-1, Du145, et al.^14^^,^^16^^,^^17^^,^^22^^,^^24^^-^^27^ There was no significant difference between weak positive and absent expression of Dab2 in human cancer cells (OR = 1.04, 95% CI: 0.66 - 1.65, *P* = 0.85; I^2 ^= 39.9%, *P *_heterogeneity_ = 0.11) (Fig. 2B). Results of the Pearson χ^2^ test revealed that abnormal expression of Dab2 was not significant correlated with the types of cancers from which cancer cell lines originated (χ^2^ = 3.23, *P *= 0.36).

**Fig.3:**
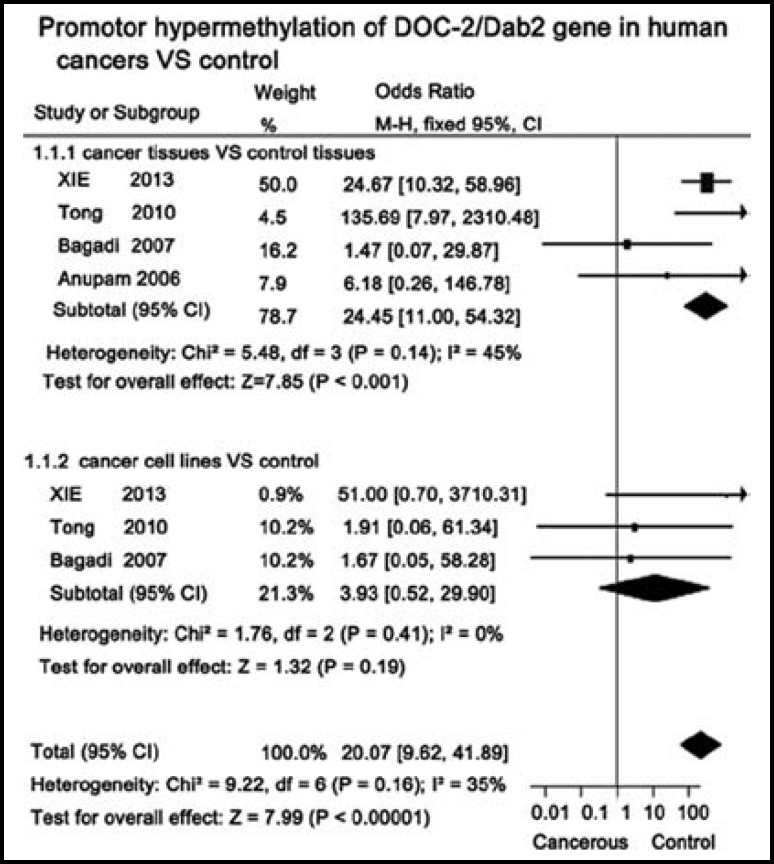
Forest plots of the meta-analysis of promotor hypermethylation of Dab2 in human cancers tissues and cell lines

***Reduced*** ***express*** ***ion of Dab2 was correlated with the aberrant promotor hypermethylation in human cancers: ***Bisulfite sequencing and methylation specific PCR (MSP) were employed to explore the correlations between promoter aberrant hypermethylation of *Dab2* and expressing reduction in 4 studies.^16^^,^^17^^,^^19^ Subgroup analysis was applied to discriminate the discrepancies of aberrant promoter hypermethylation of Dab2 in cancer tissues and cell lines. Results showed that ratio of *Dab2* promoter hypermethylation is 34.54% in cancer tissues which Dab2 expression are lost, and *Dab2* promoter hypermethylation might play a key role in the down-regulated expression of Dab2 in human cancer tissues (OR = 24.45, 95% CI: 11.00 - 54.32, *P* < 0.001; I^2 ^= 45%, *P *_heterogeneity_ = 0.14) (Fig.3).

***Publication bias: ***Egger test^28^ was performed to observe potential publication bias in each meta-analysis, and results showed no evidence of publication bias for each outcome: expression loss in human cancers tissues (*P*_Egger_ = 0.092), expression reduced in various cancer cell lines (*P*_Egger_ = 0.086) and aberrant promoter hypermethylation (*P*_Egger_ = 0.061).

## DISCUSSION

In this first systematic review, Dab2 expression was analyzed in approximately 789 human tumor and 432 normal tissues of 15 included papers. Some studies demonstrated that Dab2 protein was un-detectable in 70% ~ 90% human malignant cancers, including nasopharyngeal carcinomas, breast cancers, and gestational choriocarcinomas.^16^^,^^17^^,^^20^ However, other studies have suggested the weak to moderate positive immunostained of Dab2 expression in lung cancers, and ESCCs, et al.^15^^,^^19^^,^^21^ These diversities may be correlated with the tissue-specific differentially expression patterns of Dab2.^10^^,^^11^ Similarly, our previous studies on lung cancers suggest that there are different functions between p96-Dab2 and p67-Dab2 in the process of oncogenesis.^14^

Unfortunately, some weaknesses of current researches on Dab2 in cancer cell lines are identified in this meta study. Bagadi et al. reported that Dab2 was lost in all the breast cancer cell lines containing MDA-MB-231^17^; conversely, Cheong et al. hold opinions that it was weak positive not absent expression of Dab2 in MDA-MB-231.^27^ More interestingly, we and other researchers found that both lung cancer and 60% of TCC cell lines showed weak to moderate positive expression of Dab2 protein.^14^^,^^25^ Thus, there is not enough evidence to determine that whether the loss or weak positive of Dab2 expressions are analogous in all the human malignant cancer cell lines (*P* = 0.85). Furthermore, new standards must be ruled out for the evaluation of Dab2 expression in cancer cell lines in the near future.

**Table-I:** Characteristics of eligible studies for Dab2 expression in human cancers

**Country**	**The First Author ** ^Year^	**Materials**	**M** **ethods**	**QS**
USA	SC Mok ^1998^ [12]	44 ovarian cancers, 16 borderline ovarian tumors, 6 benign ovarian tumors, 13 normal human ovaries	IHC	6.5
	Vilmos Fulop ^1998^ [20]	17 partial hydatidiform moles, 25 complete moles and 11 gestational choriocarcinomas	IHC	6.0
	Elizabeth R. Smith ^2001^[22]	F9 mouse and PA-1 (human teratocarcinoma cells)	WB	5.5
	Zj Sheng ^2000^ [23]	47 paired ovarian tumor and non- tumor tissues	IHC	5.5
	Shao-Chun Wang ^2001^[24]	2 breast cancer cell lines a	IHC, WB	5.5
	DH Yang ^2002^ [13]	50 ovarian tumor and 5 non-tumor tissues	IHC	6.5
	Jian Zhou ^2005^ [25]	Seven human transitional cell carcinoma (TCC) cell lines b	WB	6.0
	Jian Zhou ^2005^[26]	7 prostatic cancer cells c, 5 normal prostatic epithelial cells d, and PZ-HPV7 cell	WB	6.5
	JoseA Karam ^2007 ^[21]	209 patients with Malignant urothelial Carcinoma of the Bladder, 9 patients with normal blader, 44 patients with Ta, Tis, or T1 UCB	IHC	7.0
China	Hong-Tao Xu ^2011^ [15]	105 lung cancer and 105 matched normal tissue samples	IHC	7.0
	Xue-Mei Xie ^2013^ [14]	100 lung cancer and paired normal tissue samples, eight lung cancer cell lines i	WB, MSP j	7.5
	Joanna H Tong ^2010 ^[16]	3 NPC cell lines f, 5 xenografts g, 46 nasopharyngeal carcinoma tissue samples.	WB, MSP k	7.5
India	S. A. R. Bagadi ^2007 ^[17]	6 breast cancer cell lines e, and MCF 10A, 54 breast cancer tissue samples	WB, MSP l	7.5
	Kumar Anupam ^2006^ [19]	50 ESCCs, 10 non-malignant esophageal mucosa, 30 hyperplasia and 15 dysplasia tissues	IHC, MSP l	7.0
Korea	Seong-Moon Cheong ^2012 ^[27]	8 cancer cell lines h and 1 human umbilical vein endothelial cell	WB	7.0

QS: Quality Score; IHC: Immunohistochemistry; WB: Western Blot analysis.

a SK-BR-3 and MDA-MB -453 cell lines.

b T24, TCC, UMUC3, WH, SWBC1, 253J, and RT4 cell lines.

C LNCaP, C4-2, p59-23 clone(13), COS cells; LAPC4, MDAPCa2a, and MDAPCa2b cell lines.

d PrEC1, PrEC2, PrEC3, SWNPC2, SWPC1, and SWPC3 cell lines.

e MCF7, T47D, MDA-MB-231, ZR-75-1, MDA-MB-157, MDA-MB-436.

f C666-1, HK1 and HONE1.

g X2117, X666, C15, C17, and X1915.

h A549 (lung cancer), SH-SY5Y (neuroblastoma), MDA-MB231 and MCF7 (breast cancer), HT1080 (fibrosarcoma), HepG2 (hepatoma), Du145 (prostate cancer), and SW480 (colon cancer).

I A549, H157, H1299, H460, LTE, SPC, BE1 and LK2.

j nest PCR primer, Forward: 5'-AAAGGTAGTTTTTTGTTTAAAGGG-3', Reverse, 5'-TAAACTTAATAA -CTCCCCCTCA-3'. MSP primers, Methylated Forward: 5'-GGATTTGTGAAACGAAGTTC-3', Reverse: 5'-CACCAACTAAAAAC-GATCG-3'; Un-methylated Forward: 5'-GGATTTGTGAAATGAAGTTT-3', Reverse, 5'-CACCAACTAAAAACAATCA -3'.

k Methylated Primer, Forward: 5'-ATTTTTCGTCGGGAGTGGTC-3', Reverse: 5'-GCAACGAATACGACGA -ACCT-3'; Un-methylated Primer, Forward: 5'-GGGAGTGGTTGTGTGGTTTT-3', Reverse: 5'-AACTTGG -GGACACCCAAA-3'.

l Primer for *DAB2 *exon1. Methylated Primer, Forward: 5’-TATTTTTCGTCGGGAGTGGTCGC-3’, Reverse: 5’-ACTAACTATTACCTCCGTAAA; UnMethylated Forward: 5’-GAATTATATTTTTTGTTGGGAGTGGT -TGT-3', Reverse: 5’-CCAACTAACTATTACCTCCATAAAACA-3’.

**Table-II:** The expression of Dab2 in five kinds of human cancerous and the corresponding Non-Cancerous tissues by immunohistochemistry

Type of cancers* Tissues				**Expression level of Dab2**			**Total**
				**High**	**Moderate**	**Weak or Negative**	
LC	Lung Tissues	Non-Cancerous	Count	59	39	7	105
			% within Tissues	56.2%	37.1%	6.7%	100.0%
		Malignant Cancerous	Count	29	50	26	105
			% within Tissues	27.6%	47.6%	24.8%	100.0%
UCB	Bladder Tissues	Non-Cancerous	Count	8	1	0	9
			% within Tissues	88.9%	11.1%	0.0%	100.0%
		LP or Benign Tumors	Count	18	26	0	44
			% within Tissues	40.9%	59.1%	0.0%	100.0%
		Malignant Cancerous	Count	52	117	40	209
			% within Tissues	24.8%	56.0%	19.2%	100.0%
GC	Trophoblast-ic Tissues	Non-Cancerous	Count	14	4	0	18
			% within Tissues	77.8%	22.2%	0.0%	100.0%
		LP or Benign Tumors	Count	8	15	19	42
			% within Tissues	19.0%	35.7%	45.2%	100.0%
		Malignant Cancerous	Count	0	4	7	11
			% within Tissues	0.0%	36.4%	63.6%	100.0%
ESCCs	Esophageal Tissues	Non-Cancerous	Count	30	5	5	40
			% within Tissues	75.0%	12.5%	12.5%	100.0%
		LP or Benign Tumors	Count	0	5	10	15
			% within Tissues	0.0%	33.3%	66.7%	100.0%
		Malignant Cancerous	Count	0	16	34	50
			% within Tissues	0.0%	32.0%	68.0%	100.0%
HOTs	Ovarian Tissues	Non-Cancerous	Count	12	1	0	13
			% within Tissues	92.3%	7.7%	0.0%	100.0%
		LP or Benign Tumors	Count	8	11	3	22
			% within Tissues	36.4%	50.0%	13.6%	100.0%
		Malignant Cancerous	Count	2	5	37	44
			% within Tissues	4.5%	11.4%	84.1%	100.0%

LC: Lung Cancers; UCB: Urothelial Carcinoma of the Bladder; GC: Gestational Choriocarcinomas;

ESCCs: Esophageal Squamous Cell Cancers; HOTs: Human Ovarian Tumors. LP: Lesion Precancerous.

Our research reveals that the promoter hypermethylation of *Dab2* is an important factors for the loss expression of Dab2 in human cancers tissues (OR = 24.45, *P* < 0.001). Although *Dab2 *promoter hyper-methylation have been observed in some cancer cells, there are still few reasons to attribute down-regulated expression of Dab2 to the promoter hypermethylation unless further credible evidences emerge from other cancer cells (*P* = 0.19).

In conclusion, frequent loss expressions of Dab2 are common in human malignant cancer tissues, and significantly correlated with the promoter hypermethylation. More studies would be conducted to enhance the expression of Dab2, and eliminate the aberrant hypermethylation of *Dab2*, which would offer some potential therapeutic treatment methods for human malignant cancers.

## Author Contributions:

**ZyZ** conceived, designed and did statistical analysis & editing of manuscript

**YhC, XmX, & JjT** did data collection and manuscript writing.

**XmX** did review and final approval of manuscript. **XmX** was responsible for planning the study.
